# A “groovy” diagnosis: Eosinophilic fasciitis

**DOI:** 10.1002/ccr3.3128

**Published:** 2020-07-15

**Authors:** Peter Korsten

**Affiliations:** ^1^ Department of Nephrology and Rheumatology University Medical Center Göttingen Göttingen Germany

**Keywords:** eosinophilia, eosinophilic fasciitis, immunosuppression, localized scleroderma

## Abstract

Clinical examination can be the key to pursuing a diagnosis, even in rare diseases. The “groove sign” is a typical finding in eosinophilic fasciitis and can be elicited by elevation of the arm to allow for venous return with visible “grooving” of the veins due to skin thickening.

## Graphical abstract

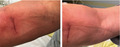

## 1 INTRODUCTION

**Figure 1:**
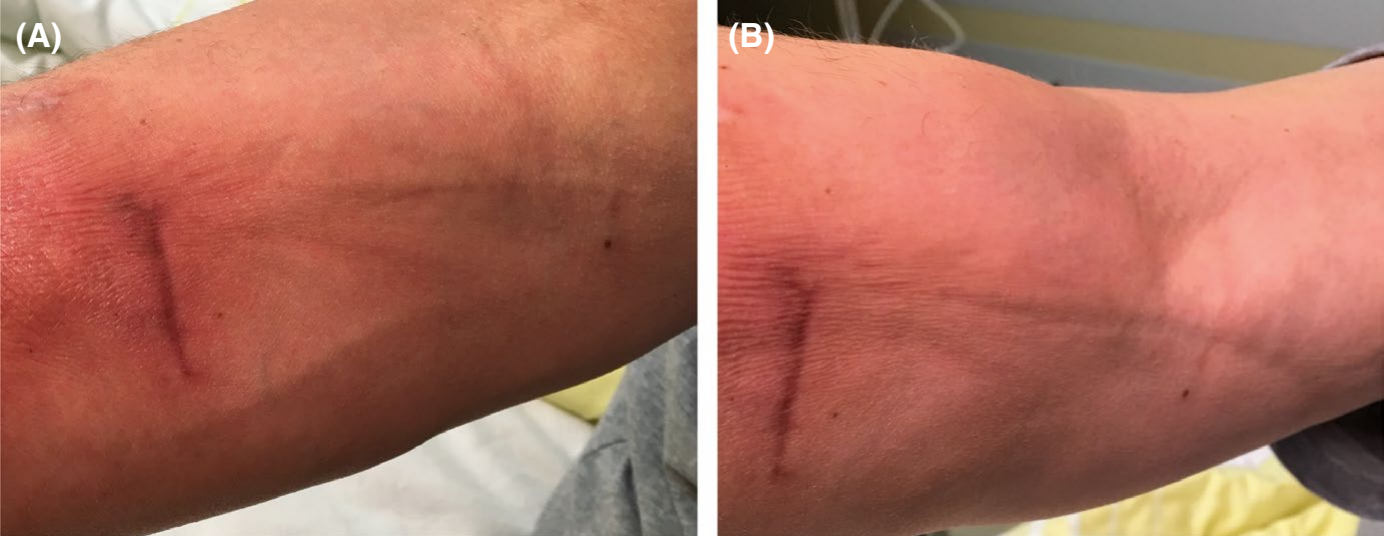
Demonstration of the groove sign. Visible veins of the forearm before elevation of the arm above the heart level (A). Retracted veins with groove‐like appearance after arm elevation (B)

A 55‐year‐old male patient presented with a 10‐month history of discoloration and skin thickening of both forearms and shins. His past medical history included papillary thyroid carcinoma in remission but was otherwise unremarkable. He reported no other symptoms. On clinical examination, the range of motion of his elbow joints was restricted due to skin tightening. Upon elevation of his arms above the heart level, there were visible retractions of the veins (“groove sign”) (Figure 1A and B). Laboratory evaluation revealed eosinophilia of 13% and elevated erythrocyte sedimentation rate. Tests for autoantibodies were negative. A full‐thickness biopsy of the skin revealed lymphocytic infiltration and fasciitis, consistent with eosinophilic fasciitis (EF, Shulman syndrome). Treatment with prednisone and methotrexate was initiated and led to a substantial improvement after 2 years of follow‐up. EF is a rare disorder with an unknown prevalence^1^ and is part of the localized scleroderma spectrum. Patients usually present in their fourth to the fifth decade with pruritus and skin thickening. A full‐thickness biopsy is required for the diagnosis. The “groove sign” is a classical clinical finding and, if present, should raise suspicion for a diagnosis of EF. Treatment with prednisone, methotrexate, or other immunosuppression usually leads to significant improvement^2^.

## CONFLICT OF INTEREST

None declared.

## AUTHOR CONTRIBUTIONS

PK treated the patient, created the figure, and wrote the manuscript.

## ETHICS STATEMENT

Written informed consent was obtained for use of the clinical images.
